# Low-cost molecular methods to characterise gastrointestinal nematode co-infections of goats in Africa

**DOI:** 10.1186/s13071-023-05816-y

**Published:** 2023-06-29

**Authors:** Paul M. Airs, Javier Ventura-Cordero, Winchester Mvula, Taro Takahashi, Jan Van Wyk, Patson Nalivata, Andrews Safalaoh, Eric R. Morgan

**Affiliations:** 1 School of Biological Sciences, Queen’s University Belfast, Belfast, Antrim UK; 2 Animal Science Department, Lilongwe University of Agriculture and Natural Resources (LUANAR), Lilongwe, Malawi; 3 Net Zero and Resilient Farming Directorate, Rothamsted Research, Okehampton, Devon UK; 4 Bristol Veterinary School, University of Bristol, Langford, Somerset UK; 5 Department of Veterinary Tropical Diseases, University of Pretoria, Pretoria, South Africa

**Keywords:** Low-resource, Faecal DNA, Species-specific PCR, Amplicon sequencing, High-resolution melt curve, Nemabiome, Haemonchosis

## Abstract

**Background:**

Veterinary diagnostics aid intervention strategies, track zoonoses, and direct selective breeding programs in livestock. In ruminants, gastrointestinal nematode (GIN) parasites are a major cause of production losses, but morphologically similar species limit our understanding of how specific GIN co-infections impact health in resource-limited settings. To estimate the presence and relative abundance of GINs and other helminths at the species level, we sought to develop a low-cost and low-resource molecular toolkit applied to goats from rural Malawi smallholdings.

**Methods:**

Goats were subjected to health scoring and faecal sampling on smallholdings in Lilongwe district, Malawi. Infection intensities were estimated by faecal nematode egg counts with a faecal subsample desiccated for DNA analysis. Two DNA extraction methods were tested (low-resource magbead kit vs high-resource spin-column kit), with resulting DNA screened by endpoint polymerase chain reaction (PCR), semi-quantitative PCR, quantitative PCR (qPCR), high-resolution melt curve analysis (HRMC), and ‘nemabiome’ internal transcribed spacer 2 (ITS-2) amplicon sequencing.

**Results:**

Both DNA isolation methods yielded comparable results despite poorer DNA purity and faecal contaminant carryover from the low-resource magbead method. GINs were detected in 100% of samples regardless of infection intensity. Co-infections with GINs and coccidia (*Eimeria* spp.) were present in most goats, with GIN populations dominated by *Haemonchus contortus*, *Trichostrongylus colubriformis*, *Trichostrongylus axei*, and *Oesophagostomum columbianum*. Both multiplex PCR and qPCR were highly predictive of GIN species proportions obtained using nemabiome amplicon sequencing; however, HRMC was less reliable than PCR in predicting the presence of particular species.

**Conclusions:**

These data represent the first ‘nemabiome’ sequencing of GINs from naturally infected smallholder goats in Africa and show the variable nature of GIN co-infections between individual animals. A similar level of granularity was detected by semi-quantitative PCR methods, which provided an accurate summary of species composition.

Assessing GIN co-infections is therefore possible using cost-efficient low-resource DNA extraction and PCR approaches that can increase the capacity of molecular resources in areas where sequencing platforms are not available; and also open the door to affordable molecular GIN diagnostics. Given the diverse nature of infections in livestock and wildlife, these approaches have potential for disease surveillance in other areas.

**Graphical Abstract:**

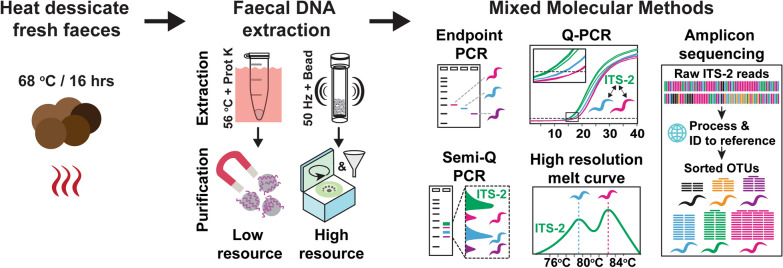

**Supplementary Information:**

The online version contains supplementary material available at 10.1186/s13071-023-05816-y.

## Background

Within veterinary parasitology, molecular diagnostics have largely been limited to research environments, but falling costs of DNA sequencing technologies give promise for molecular diagnostics to complement or replace traditional morphological approaches as our understanding of parasite genomics improves [1–3]. Molecular techniques are critical for the advancement of livestock sciences and can facilitate improved monitoring of parasitic diseases and their drug resistance traits that threaten livestock systems and food security worldwide [4–6]. While a number of molecular approaches have been developed in recent years to monitor parasites of veterinary significance [2, 3, 7, 8], there has been limited optimisation of approaches amenable to low-resource areas owing largely to the lack of capacity. However, the advent of COVID-19 has resulted in a push to overcome logistical barriers limiting molecular techniques in lower-resource areas and has increased the capacity for molecular diagnostics worldwide in health settings [9–11]. As such, the development of approaches to monitor veterinary parasites at low cost and with reduced resources has potential for application worldwide. Currently, the utility of molecular techniques to detect gastrointestinal nematode (GIN) parasites is also limited in commercial livestock settings, but may become more essential as tropical species, notably the GIN *Haemonchus contortus*, spread under climate change [12–14].

In low-income rural areas, livestock holdings are often inextricably linked to livelihoods. For instance, in rural Malawi, goat smallholdings buffer against food insecurity and provide supplemental income [15–17]. However, goat smallholdings also suffer from animal losses and reduced productivity driven by co-infections with a mix of gastrointestinal parasites including numerous GIN species [17–21]. GINs play a major role in production losses of small ruminant livestock systems worldwide [22, 23], causing gastroenteritis and stress on the goat immune system, resulting in reduced weight gain, weight loss, anaemia, or death [24–26]. While the impact of individual species is known, the prevalence and relative abundance of these species is poorly described in smallholder systems. Additionally, the population dynamics of GIN co-infections and their impact on individual goats have yet to be determined. This is partly due to the fact that in low-resource settings like those of rural Malawi, pragmatic and accessible tools such as the Five Point Check^©^ (FPC) are used to measure signs of GIN infections [27–29], but these do not measure infection intensity, the presence of specific species, or drug resistance traits. Beyond the FPC, faecal egg counts (FECs) can be performed on fresh faecal samples [30, 31]; however, more dominant and pathogenic species of strongyles, including *H. contortus*, *Oesophagostomum columbianum*, *Trichostrongylus colubriformis*, and *Trichostrongylus axei*, are not readily distinguishable from each other or from less pathogenic species by egg morphology alone. Since the presence of different GIN species can alter infection outcomes, as well as results of drug efficacy testing, it is useful to identify both the different species present and their relative abundance in co-infections. This is possible by morphological identification of third-stage larvae (L3). However, this approach is time-consuming and labour-intensive, requires specific expertise, and critically can only be performed on fresh faecal material which cannot be easily biobanked, especially in tropical conditions.

Molecular approaches offer an alternative to determine GIN species from biobanked material with preserved DNA. Faecal DNA extractions have been shown to predict infection burden [32–34] and can be more sensitive than FECs for detection and quantitation of GIN infections [35]. GINs can be identified to species by a number of methods including standard polymerase chain reaction (PCR) [32, 33, 36, 37], loop-mediated isothermal amplification (LAMP) [38, 39], and high-resolution melt curve (HRMC) analyses [32, 40–42] among others. However, quantification of GIN populations has so far relied on advanced and costly methods such as real-time PCR [34, 35, 43, 44], digital PCR [45, 46], or internal transcribed spacer 2 (ITS-2) deep amplicon sequencing [47–51]. While quantitative approaches are robust, there is potential to utilise standard PCR techniques to estimate relative GIN species abundance at a significantly reduced cost. Studies investigating the quantification of GINs by standard PCR techniques typically involve artificial infections and published studies of PCR or HRMC applications in rural goats in real-world settings are lacking.

To reduce the cost, effort, and expertise required to profile the relative abundance of different GIN species present in infected individual goats, we sought to probe low-cost molecular methods using a simple heat desiccation biobanking method on goat faecal pellets and validate findings against real-time PCR [34, 35, 43, 44] and nemabiome deep-amplicon sequencing techniques [47–51].

## Methods

### Faecal sample collection and demographics

With permission from smallholder farmers in the Dedza region of central Malawi (Mkwinda and Chinkowe Group Village Head areas) a total of 47 goats were examined across 15 farms (range = 1–6 goats per farmer, median = 3) on February 4–5, 2020. To ensure goats in the study were given proper care, all goats included in the study were assessed by the Five Point Check^©^ [52] and were provided anthelmintic intervention if in poor condition. Goat age was estimated by dental examination and weight was estimated by calibrated goat girth weigh tape (Coburn, cat. 44558). Pregnancy and lactation status data were collected from farmers. After examination, faecal pellets were collected by stimulating the anal sphincter using gloved hands with lubricant. Faecal samples from individual goats were immediately transferred to plastic bags (1 bag per goat) and placed in a cooler box with ice until processing.

### Five Point Check^©^ (FPC) scores

**Fig. 1:**
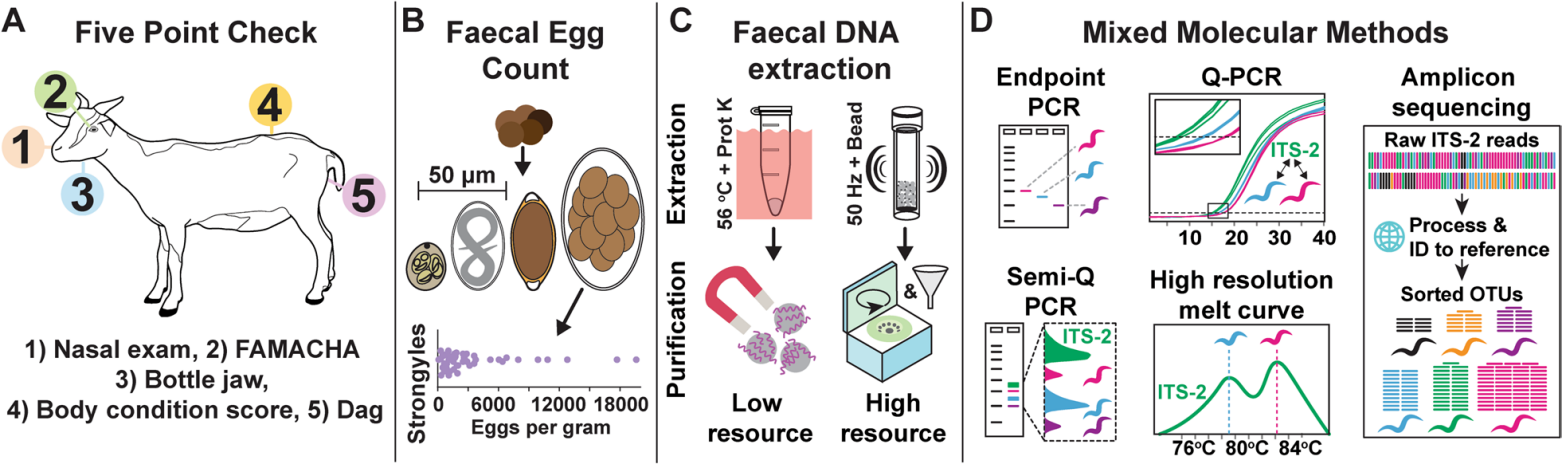
Study design. Health status and GIN infections were assessed by **A** the Five Point Check^©^ score for signs of disease including (1) presence of nasal discharge, (2) anaemia measured by a FAMACHA card, (3) presence of pitting oedema or bottle jaw, (4) body condition scoring, and (5) presence of dag or scour. **B** Direct faecal samples from individual goats were used for faecal egg counts to detect eggs of *Strongyloides*, strongyle, or *Trichuris* nematodes, or coccidial oocysts. **C** The same faecal samples from part B were desiccated and subjected to DNA extraction using a low-resource proteinase K and magbead system or a high-resource bead homogenisation and filter centrifugation system. **D** Extracted DNA was assessed by species-specific molecular methods on all or a subset of samples to detect the presence and relative abundance of GIN species. *Worm symbol* species-specific nematode identities, *ITS-2* pan-nematode region of the internal transcribed spacer 2

Baseline goat health was measured by the FPC as described previously [27]. The score includes examination of (1) nasal discharge, (2) anaemia by conjunctival examination using a Faffa Malan color chart (FAMACHA), (3) presence of pitting oedema or ‘bottle jaw’, (4) body condition score (BCS), and (5) presence of absence of scour or dag, i.e., perineal faecal staining (Fig. 1A). Scores are shown in Additional file 1: Table S1.

### Faecal egg counts (FEC)

Counts of egg density using the McMaster technique and morphological identification of egg types were performed per goat with 2 g of fresh faeces (< 24 h after collection) ground through a coarse sieve with a spoon into 28 ml of 1.2 g specific gravity sugar flotation solution (1290 g sucrose/1 l water). Two millilitres of the middle of the flotation solution was checked by McMaster slide to assess eggs per gram (EPG) for strongyle, *Strongyloides*, and *Trichuris* nematode eggs by morphology, in addition to qualitative coccidia counts. Coccidial oocysts per gram (OPG) were defined as follows: 0 = 0, 1 = 1–300, 2 = 300–1000, 3   ≥ 1000. Raw data are presented in Additional file 1: Table S1.

### Faecal DNA preservation

At the same time as FECs, ~ 0.3 g of fresh faecal material per goat (typically one faecal pellet) was picked by single-use toothpicks and wrapped in pre-cut foil to avoid cross-contamination. A single pellet was chosen because it is convenient to sample and limits the amount needed from voided samples given the limited total volume available and other demands on it such as for FEC, and because of the likelihood that at high egg densities, the main species present are well represented in this volume. Samples were labelled and preserved by oven drying at 68 °C for 24–36 h and then stored in sealed desiccant jars with silica beads until extraction.

### DNA extraction

Two methods were tested, with modifications (illustrated in Fig. 1): a ‘low-resource’ kit (MagaZorb^®^ DNA Mini-Prep Kit, cat. MB1004, Promega, WI, USA) with an accompanying magnetic separation rack (cat. CD4002, Promega, WI, USA), and a ‘high-resource’ kit (Quick-DNA Fecal/Soil Microbe Miniprep Kit, cat. D6010, Zymo, CA, USA). Both were performed according to the manufacturers’ instructions, with minor variations. For the MagaZorb® DNA Mini-Prep Kit, 0.027–0.153 g of dried faeces from 13 samples was subjected to extraction with extended lysis (30 min instead of 10 min) along with an additional wash buffer step and a single centrifugation step (10,000*g* for 1 min) after elution of DNA to reduce carryover debris. The Quick-DNA Fecal/Soil Microbe Miniprep Kit was performed according to the manufacturer's instructions with 0.038–0.13 g of faecal tissue across 34 samples. Samples were ground by mortar and pestle and subsequently homogenised for 2 × 3 min cycles at 50 Hz in a TissueLyser LT (cat. 69980, Qiagen, Germany). For most samples, homogenisation efficiency was measured by 600 nm absorbance (A:600). A:600 was calculated after the addition of genomic lysis buffer (Zymo kit, with mixed genomic lysis buffer and bashing bead buffer blank) or binding buffer (for the Promega kit with mixed lysis buffer and binding buffer blank). DNA quality and yields were calculated by a NanoDrop 1000 Spectrophotometer (Thermo Scientific) with three readings taken per sample. All samples were standardised to 4.8 ng/μl (lowest sample average) in nuclease-free H_2_O in a 48-well plate array (including a no-template control well). DNA input weight, yield, purity, and A:600 readings are shown in Additional file 1: Table S1.

### Endpoint PCR

Species-specific PCR targeting *H. contortus* and *Haemonchus placei* [36, 53], *T. axei* and *T. colubriformis* [36], *Fasciola gigantica* and *Fasciola hepatica* [54], *O. columbianum* [55], *Bunostomum trigonocephalum* [36], *Chabertia ovina* [36], *Teladorsagia circumcincta* [36, 56], *Nematodirus spathiger* (this study), and the genus *Eimeria* [57] using primers from cited work (Additional file 2: Table S2). The *T. axei* primer can also be used for *Trichostrongylus vitrinus* PCR, but no *T. vitrinus* was identified in this study. GoTaq^®^ G2 Hot Start Taq Polymerase (cat. M7405, Promega, WI, USA) was used for all PCR reactions according to the manufacturer's instructions with 0.5 μl of DNA template per 12.5 μl PCR reaction. Cycling conditions included an initial denaturation (95 °C/2 min), 35 cycles (detailed per primer set in Additional file 2: Table S2), and final extension (72 °C/5 min) on a MiniAmp™ Plus Thermal Cycler (Applied Biosystems™, cat. A37835).

### Semi-quantitative PCR

Detection of the strongyle species *H. contortus*, *T. colubriformis*, and *T. axei* was performed with pan-nematode ITS-2 primers used as an internal reference (Additional file 2: Table S2). For singleplex PCR, *O. columbianum* was also detected with a specific primer set (Additional file 2: Table S2). PCR was performed as described above but with 4.8 ng of DNA template in 50 μl reaction volumes. For multiplex PCR, 2 μl Tcol R1, 2 μl Tvit F1, 2 μl Hcon F3, 4 μl ITS F, and 2 μl ITS R primers were used per reaction at recommended concentrations (Additional file 2: Table S2). Band intensities were assessed at 25, 29, and 35 cycles, with 29 cycles selected for singleplex PCR and 35 cycles selected for multiplex PCR.

### Quantitative PCR (qPCR) and HRMC analyses

Twenty nanograms of template DNA was added per 15 μl reaction (SYBR™ Select, Thermo Scientific, cat. 4472937) with 0.6 μl of *H. contortus*, *T. colubriformis*, or ITS-2 primers (10 μM stock) at the recommended concentration (Additional file 2: Table S2). Cycling was performed in strip tubes (Cleaver Scientific, cat. RGCS-250) on a Rotor-Gene Q (Qiagen) instrument with the following conditions: UDG activation (50 °C/2 min), initial denaturation (95 °C/2 min), amplification (40 cycles of 95 °C/15 s, 54 °C/15 s, 72 °C/30 s), and melt curve (0.5 °C from 70–95 °C). All reactions were performed in triplicate with average Ct values and melt curve peaks used for analysis. For qPCR analysis, the relative abundance of *H. contortus* versus *T. colubriformis* was calculated as a percentage difference from the average fold-change compared to the ITS-2 internal control (ΔC_t_ method). HRMC analysis was performed on melt curve average peaks from triplicate ITS-2 products following qPCR.

### Gel electrophoresis relative abundance analysis

For all standard PCR analyses, 5 μl of PCR product or 5 μl ladder (PCRBIO Ladder IV, cat. PB40.14-05, PCR Biosystems) was loaded into 1.4% agarose gels using Tris–boric acid-EDTA buffer pre-cast with GelRed^®^ Nucleic Acid Gel Stain (Biotium, cat. 41003) run at 90–110 V for 30–60 min or until bands were visibly separated. Custom-designed laser-cut multichannel gel combs were produced to facilitate rapid and accurate loading of samples (Additional file 7: File S1). Tag image file format (tiff) images were taken under a gel imager with automated exposure and analysed in FIJI [58]. Images were inverted, saturation thresholds were automatically normalised with the brightness and contrast tool, and the background was removed with a 50-pixel rolling-ball radius. PCR product band intensities were then measured horizontally (endpoint PCR scans of entire gel row) or vertically per band (singleplex and multiplex) as demonstrated in Fig. 3A–C using the GelAnalyzer tool. Relative abundance was calculated as the percent of total peak area among specific species identified with ITS-2 bands used as an internal reference only.

### Nemabiome ITS-2 deep amplicon sequencing

To assess for GINs across goat samples, 4.8 ng from each DNA extraction was pooled and mixed by vortex (*n* = 47). In addition, eight individuals were selected as case studies for cross-comparison with PCR findings. Amplicon sequencing was performed according to published protocols [47] available at https://nemabiome.ca with modifications. Twenty nanograms of template DNA was added per 30 μl PCR reaction (Q5^®^ High-Fidelity DNA Polymerase, NEB, cat. M0491L) with a mix of ITS-2 primers containing 0–3 ‘N’ bases and an Illumina adapter (10 μM stock) used at recommended concentrations. Cycling included 35 cycles as described with primer details in Additional file 2: Table S2 with a 98 °C/2 min initial denaturation and 72 °C/2 min final extension. PCR products were assessed by 1.2% gel electrophoresis as described above and purified (Wizard^®^ SV Gel and PCR Clean-Up System, Promega, cat. A9281). Library prep and sequencing were performed via NGSelect Amplicon 2nd PCR service (Eurofins Genomics) on the MiSeq Illumina platform with the 2 × 250 v2 Reagent Kit (Illumina) with resulting metrics from trimmed reads (by Cutadapt [59]) shown in (Additional file 3: Table S3). An average read depth of 242,720 high-quality reads (range 201,800–299,860) was achieved.

### Bioinformatics analysis

Run quality was assessed by DADA2 [60] and phyloseq [61]. Phyloseq was used for comparison purposes only. Taxonomic sequence assessments were made in Mothur version 1.48.0 (https://mothur.org/) [62] as described previously [47] using pipeline information at nemabiome.ca. Trimmed reads were merged into contigs 200–450 base pairs in length and reads containing ambiguities removed. Contigs were aligned to a curated nematode ITS-2 ribosomal DNA (rDNA) database [63] with a 90% sequence similarity cut-off. Non-aligned sequences were classified by the *k*-nearest neighbor algorithm where *k* = 3, with remainders classified to the next taxonomic level (i.e., genus level if species matching fails). PCR bias correction factors were applied to raw reads including *T. axei* (0.9647), *T. colubriformis* (1.0239), and *H. contortus* (0.6970) based on recommended protocols at nemabiome.ca from prior optimisations [49].

### Data presentation and statistical analyses

Data were tabulated in Microsoft Excel, with all statistical tests and graphical outputs generated with R (version 4.0.4 ‘Lost Library Book’) or GraphPad Prism (version 9.0.0 for Windows, GraphPad Software, San Diego, CA, USA). Illustrations and figure layouts were generated in Adobe^®^ Illustrator 2022 (Adobe Inc.). All cost data are shown in British pounds (£) and are derived from prices paid for reagents at the time of the study.

## Results

### Baseline goat health assessments

**Fig. 2:**
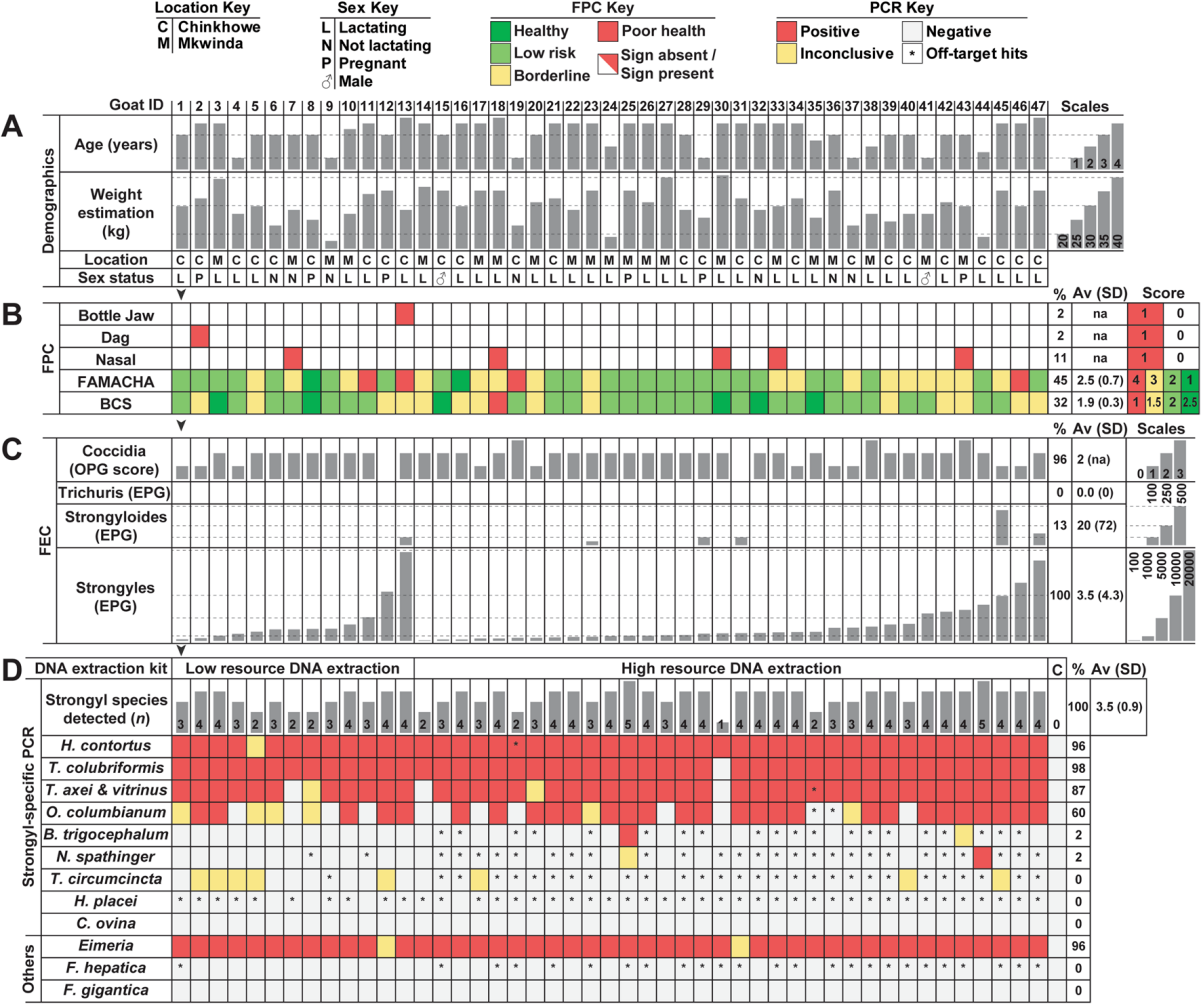
Individual goat health variation and co-infection burden. Forty-seven local breed goats assessed to determine **A** baseline goat demographics, **B** health as measured by the Five Point Check^©^ with percentage of at-risk individuals, **C** faecal egg counts of eggs per gram (EPG) or oocysts per gram (OPG), with OPG defined as 0 = 0, 1 = 1–300, 2 = 300–1000, 3 =  > 1000, and **D** presence of nematode and other helminths in faecal DNA by endpoint PCR with species-specific primers. Five Point Check^©^ scores were recorded as binary values (0 = healthy, 1 = needs intervention) or standardised scales FAMACHA (1/2 = healthy, 3 = borderline, 4 = needs intervention), BCS (2.5–2 = healthy, 1.5 = borderline, 1 = needs intervention). Goat IDs are stacked into contiguous columns for parts A–F (black arrowheads are shown for representation). Percentages from parts B–D are detection rates across specimens. For part D, presence was determined by band intensity as viewed by eye with faint bands deemed inconclusive and off-target hits determined by the presence of bands present outside of the expected size range for the target. na = not available

The relationship between health, GIN burden, and GIN species, was assessed by measuring signs of disease using the FPC, FECs, and by faecal DNA mixed molecular methods (Fig. 1). In total, 47 goats were assessed, ranging in age, weight, sex, and pregnancy status across two village areas (Fig. 2A). FPC scores taken from individual goats included occasional instances of bottle jaw, nasal discharge and dag; and variable health status based on BCS (median = 2, mean = 1.89) and FAMACHA score (median = 2, mean = 2.49), indicative of GIN infections causing disease in many individuals (Fig. 2B). Overall, 10 goats were classified as unhealthy at the time of inspection, 16 in borderline health, and 21 healthy.

FECs identified a broad range of strongyle eggs (range 100–19,600, mean = 3457, SD = 4329 EPG) and high coccidial oocyst counts on average (median = 300–1000 OPG). Overall there were very low levels of *Strongyloides* (median = 0 EPG) and no *Trichuris* found (Fig. 2C).

### Testing low-resource DNA preservation and DNA extraction methods

To enable molecular determination of GIN populations in lower resource settings, we tested a low-cost desiccation approach to preserving faecal DNA by drying ~ 0.3 g of faeces > 16 h at 68 °C. Desiccation preserves DNA in faeces [64] eliminating the need for chemicals or cold-chain infrastructure, but the impact of desiccation on GIN egg DNA is unknown. Bulk faecal DNA was purified by a ‘low-resource’ magbead or a ‘high-resource’ column DNA extraction kit designated due to differences in the cost of equipment and reagents as well as the need for electricity (Additional file 4: Table S4). The low-resource kit requires little to no electricity since the only heating step can be performed with a flame and thermometer, while a magnetic rack is used for cleaning and purification steps. The high-resource column purification kit is approximately 3.63 times more expensive per reaction and requires more expensive equipment including a mechanical bead-beater and a high-speed centrifuge (Additional file 4: Table S4). There was no difference in overall DNA yield between the methods (Additional file 8: Fig. S1A), but the low-resource method resulted in lower DNA purity (Additional file 8: Fig. S1B). The low-resource method also required a short low-speed centrifugation step in some samples to remove carry-over PCR inhibitors that blocked detection of GIN DNA in samples with more faecal contamination (Additional file 8: Fig. S1C, D). However, the coloration of faeces during extraction (a measure of homogenisation and initial composition), as measured by spectrometry (A:600 nm), did not correlate to DNA yield (Additional file 8: Fig. S1E).

### GIN and other helminth species identification by species-specific PCR

Both the low-resource and high-resource methods of extraction were capable of identifying key GIN species by Endpoint PCR (Fig. 2D). Four of the nine strongyle species tested were identified in the majority of samples, with near ubiquitous prevalence of *H. contortus* (96%) and *T. colubriformis* (98%). Some species resulted in spurious off-target hits suggesting other similar species may be present, but this could also be due to random off-target hits within faecal material (Fig. 2D). Among non-strongyle PCR tests, *Eimeria* was identified in all samples where coccidial oocysts were detected by FEC (see Fig. 2C, D), but neither *F. hepatica* nor *F. gigantica* was reliably identified. This may be due to low egg counts typical of these parasites in faeces, or these goats may not carry patent infections.

For strongyle GIN species, the infection intensity (as measured by FEC) was weakly correlated to the number of GIN species detected when using endpoint PCR as well as singleplex semi-quantitative PCR (Additional file 8: Fig. S2A, B). Critically, the detection of parasites by standard endpoint PCR was not significantly different between the low-resource (average 3.15 ± 0.8 species detected) and high-resource (average 3.55 ± 0.85 species detected) DNA extraction methods (*t*-test, *P* = 0.14). This result did not change when normalising GIN detection to infection intensity (Additional file 8: Fig. S2C). Overall, generalised correlations comparing the detection of GIN species with the strongyle FEC identified significant co-occurrence of *T. axei* alongside *T. colubriformis* and *O. columbianum* (Additional file 8: Fig. S2D). Only *O. columbianum* was significantly more likely to be identified in individuals with higher FECs (Additional file 8: Fig. S2D).

### Relative quantification of GIN co-infections by low-cost PCR methods

**Fig. 3:**
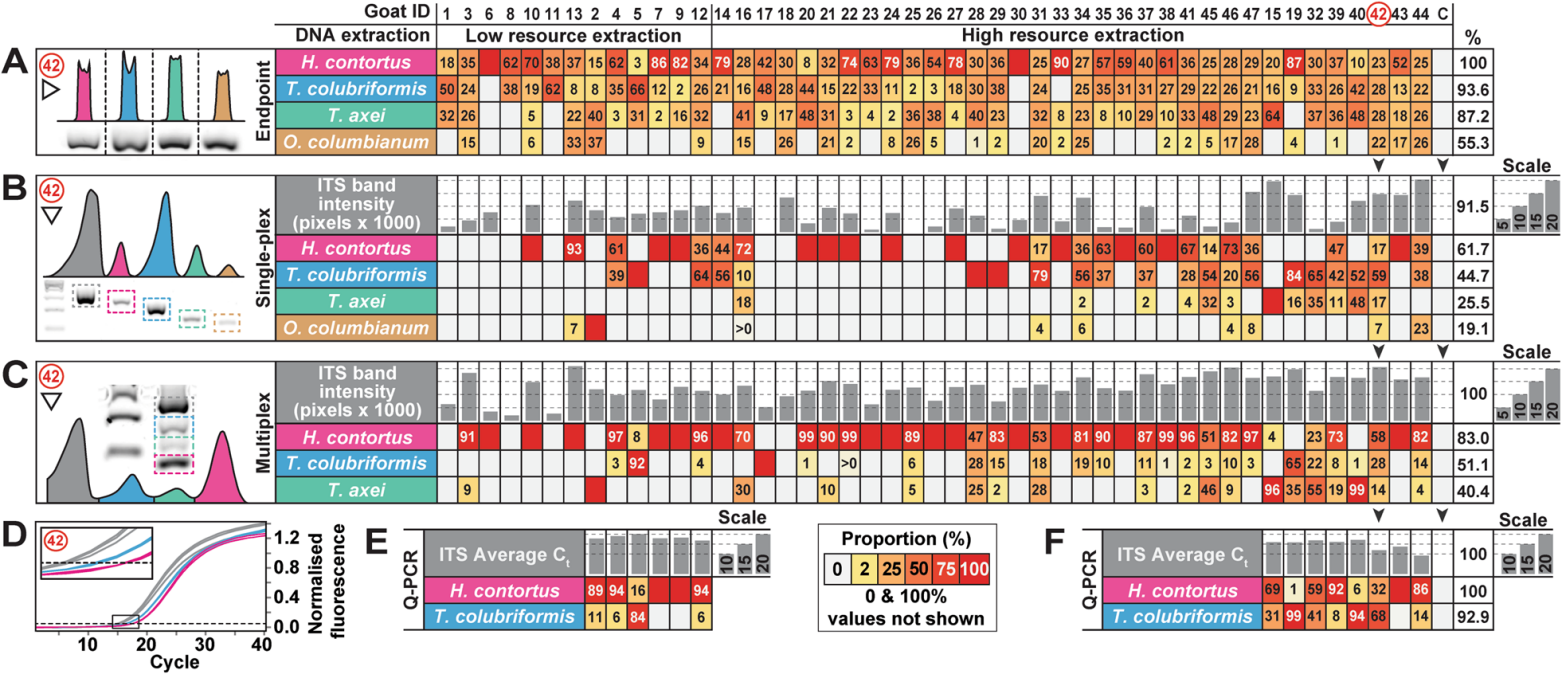
Cross-validation of PCR methods to determine GIN infection species composition. PCR methods tested on faecal DNA extractions from Goat IDs 1–47 and a negative control = C, subjected to low-resource or high-resource extraction methods. Relative abundance analyses performed by **A** endpoint 35-cycle PCR with unbalanced DNA, **B** singleplex 29-cycle semi-quantitative PCR with 4.8 ng standardised input DNA, **C** Multiplex 35-cycle PCR with 4.8 ng standardised input DNA, and **D–F** qPCR with standardised 20 ng input DNA. Band intensities determined from normalised gel images for A-C and normalised fluorescence intensity from part D with specimen 42 shown for representation. Direction of band intensity scanning shown by white arrowheads. Goat IDs are stacked into contiguous columns for parts A–F (black arrowheads shown for representation)

The species composition of GIN infra-populations was assessed using several low-cost PCR techniques as compared to qPCR (Fig. 3; Table 1). Of the different PCR methods tested, multiplex semi-quantitative PCR was the most economical and the highest throughput capable of detecting the relative abundance of three GIN species for one-third of the cost of singleplex PCR and 22.5 times less than qPCR methods (Table 1).

**Table 1:** Costs of molecular methods tested

Method	Species tested	Targets/RXN (reaction)	Replicates/RXN	Thermocycler	Throughput/run^a,c^	Cost/RXN ^b^	Cost/sample^b,c^	Cost/run^b,c^
Endpoint PCR	12	1	1	96-well MiniAmp Plus	32	£0.21	£0.63	£20.16
Singleplex PCR	4	1	1	96-well MiniAmp Plus	32	£0.21	£0.63	£20.16
Multiplex PCR	3	3	1	96-well MiniAmp Plus	96	£0.21	£0.21	£20.16
qPCR	2	1	3	72-tube Rotor-Gene Q	8	£0.53	£4.73	£37.80
HRMC	Various	Various	3	72-tube Rotor-Gene Q	24	£0.53	£4.73	£37.80

Reagent costs for methods trialled in the studies listed include the number of species targets and the throughput using equipment listed in the table. Consumables and equipment costs such as pipette tips and labour costs are not included

^a^Number of samples processed per run when testing for three species, excluding controls

^b^Cost of PCR reagents as described in the Methods section

^c^Cost calculated to check for three species per sample

The relative abundance of the most commonly identified GIN species was assessed by PCR product band intensity on agarose gels, including endpoint PCR (Fig. 3A), singleplex semi-quantitative PCR (Fig. 3B), and multiplex semi-quantitative PCR (Fig. 3C) techniques. Singleplex PCR and multiplex PCR methods were compared to pan-nematode ITS-2 PCR product band intensities, which varied between samples (Fig. 3B, C). *Haemonchus contortus* was the most prevalent species identified across all semi-quantitative PCR methods and was also the dominant species in the majority of samples. However, samples differed in the relative abundance of different species identified, and cases of *T. colubriformis* dominance over *H. contortus* were generally concordant between the two semi-quantitative PCR methods.

**Fig. 4:**
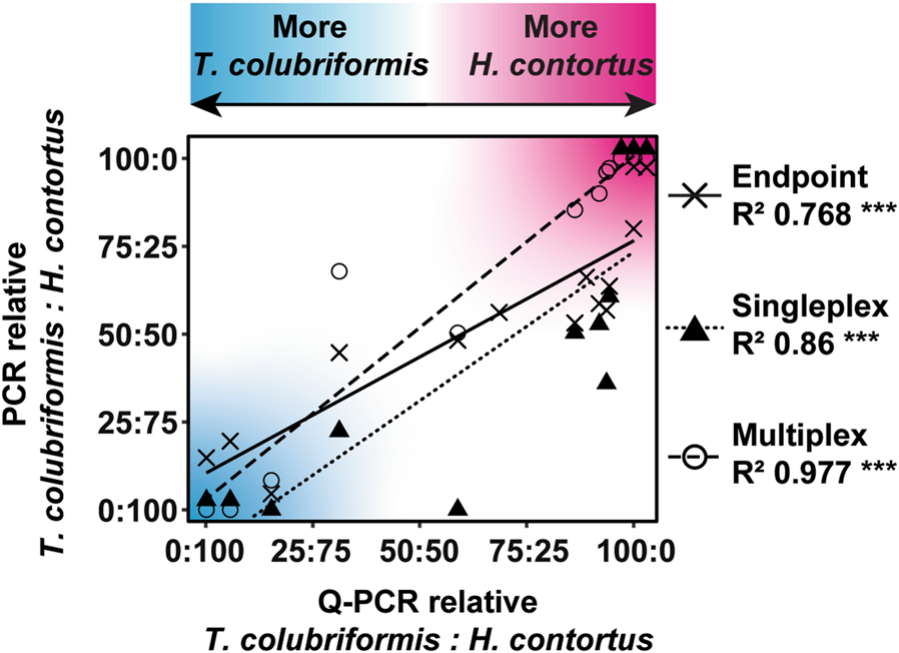
Cross-validation of PCR techniques. Regression cross-validation of standard PCR approaches versus qPCR using *H. contortus* versus *T. colubriformis* relative abundance. *P < 0.05, **P < 0.01, ***P < 0.001

To test whether semi-quantitative methods were accurate, qPCR was performed on a representative subset of samples to determine the relative abundance of *H. contortus* versus *T. colubriformis* as these two species predominated in almost all individuals (Fig. 3D, F). The relative abundance of parasite DNA determined by semi-quantitative PCR methods was cross-validated against qPCR findings, focusing on *H. contortus* and *T. colubriformis* (Fig. 4A). All of the standard PCR methods significantly correlated to qPCR findings, including the endpoint PCR method, indicating that even non-quantitative PCR may provide a generalised view of the relative abundance among different GIN species. Of these methods, the multiplex method was the best fit compared to qPCR (Fig. 4A) but also suffered from drop-out, failing to identify some species in some samples compared to the endpoint method.

The relative proportion of GINs across goats was assessed using the endpoint PCR method, identifying the most abundant species to be *H. contortus* (51.2% of individual goats), *T. colubriformis* (39.5%), *T. axei* (2.3%)*,* and *O. columbianum* (2.3%) across the dataset. These results were similar to singleplex and multiplex PCR results, although proportions differed in terms of PCR product band intensity (see Fig. 3A–C).

### Assessing HRMC from faecal DNA

**Fig. 5:**
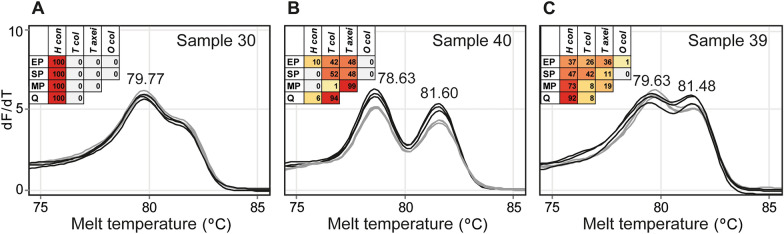
High-resolution melt curves with pan-nematode ITS-2 primers. Example samples include **A** a *H. contortus*-dominant infection with a single peak at 79.77 °C ± SD 0.04 °C, **B** a *Trichostrongylus*-dominant infection with peaks at 78.63 °C ± SD 0.027 °C and 81.6 °C ± SD 0.052 °C, and **C** a mixed infection with a *H. contortus*-associated peak at 79.63 °C ± SD 0.113 °C and a second *Trichostrongylus*-associated peak at 81.48 °C ± SD 0.04 °C. Insets show relative species abundance (as percentage) from *EP*  endpoint PCR, *SP* singleplex PCR, *MP* multiplex PCR, and Q qPCR analyses where *H con*  *H. contortus*, *T col*  *T. colubriformis*, *O col*  *O. columbianum*

To go beyond species-specific PCR to predict predominant GIN species in a non-target-specific manner, we utilised pan-nematode ITS-2 primers to produce a PCR product pool for all target species (see Additional file 2: Table S2). In this instance, HRMC can present a means to assess specific targets by their relative peaks, as has been demonstrated for other helminths [40, 41]. However, this technique has not been tested in the context of faecal DNA screening, or in conditions where unknown co-infections occur. A subset of 15 samples revealed distinct melt curve patterns in cases of *H. contortus*-dominant versus *Trichostrongylus*-dominant infections (Fig. 5; Additional file 8: Fig. S3). *Haemonchus contortus* was associated with a peak at ≈79.76 °C (see Fig. 5A) while *Trichostrongylus* species were associated with peaks at ≈78.59 °C and ≈81.58 °C (Fig. 5B, C; Additional file 5: Table S5). Overall peaks varied by up to 0.24 °C from the average and no clear distinction between *Trichostrongylus* species could be made in this analysis. Also, peaks suffered from minor variation between replicates, limiting the utility of specific temperatures marking the presence of particular species in unknown specimens.

### Nemabiome deep amplicon sequencing from faecal DNA

**Fig. 6:**
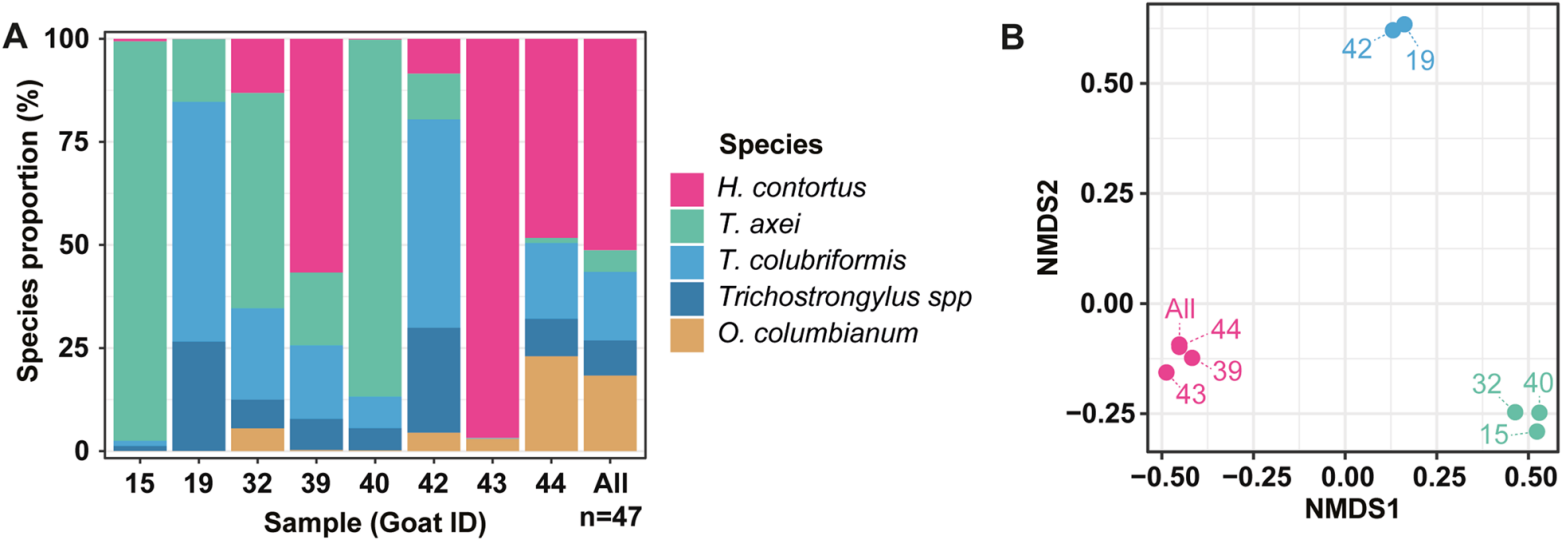
Nemabiome ITS-2 amplicon sequencing of faecal DNA. **A** Mothur-resolved proportional GIN species and unresolved *Trichostrongylus* species from select goat samples as well as pooled DNA from all goat samples. **B** Bray–Curtis non-metric multidimensional scaling (NMDS) clusters coloured by most abundant species detected by DADA2

While PCR methods can accurately determine the relative abundance of specific GIN species, the number of detectable species is limited to the number of primers or probes used. To see whether other GIN species are present in goat faecal DNA, and to measure the relative proportion of reads from each species, we turned to nemabiome deep amplicon sequencing for a subset of samples as well as a pooled sample from all 47 individuals (Fig. 6). Nemabiome reads as assessed by the Mothur pipeline revealed the four main species identified by PCR (*H. contortus*, *T. axei*, *T. colubriformis*, *O. columbianum*) to be by far the most common species detected by nemabiome representing 91.45% of reads from the pooled DNA sample (Fig. 6A). *Haemonchus contortus* was the most abundant GIN from the pooled sample representing 51.3% of reads. However, among *Trichostrongylus* species 28% of reads could not be determined to species level, accounting for 8.54% of total reads for the pooled sample. The proportion of unclassified *Trichostrongylus* species differed between samples but made just over 25% of reads in samples 19 and 42 (Fig. 6A). Classifying taxa using the DADA2 pipeline with IDTAXA resulted in all *Trichostrongylus* reads assigned to either *T. axei* or *T. colubriformis* (Additional file 8: Fig. S4A). Comparing the two pipelines revealed almost all unspeciated *Trichostrongylus* reads from Mothur to be classified as *T. colubriformis* by IDTAXA (Additional file 6: Table S6). Conversely, IDTAXA did not identify to species the majority of *Oesophagostomum* reads, which were all classified as *O. columbianum* by Mothur (Additional file 6: Table S6). IDTAXA also classified a small number of reads as *Ostertagia* and *Spiculopteragia* species (Additional file 8: Fig. S4; Additional file 6: Table S6). Overall GIN proportion differences between Mothur and IDTAXA pipelines were minimal when accounting for these differences.

Nemabiome results revealed drastic differences in species proportions between individual goats, highlighting differences in GIN infra-population composition (Fig. 6A). To assess whether co-infections follow specific signatures, we performed non-metric multidimensional scaling (NMDS) from DADA2 analyses (Fig. 6B). Despite the small number of species identified distinct clusters formed, which appeared to be determined by the most abundant species detected.

### Validating low-cost PCR methods and HRMC analyses with nemabiome results

**Fig. 7:**
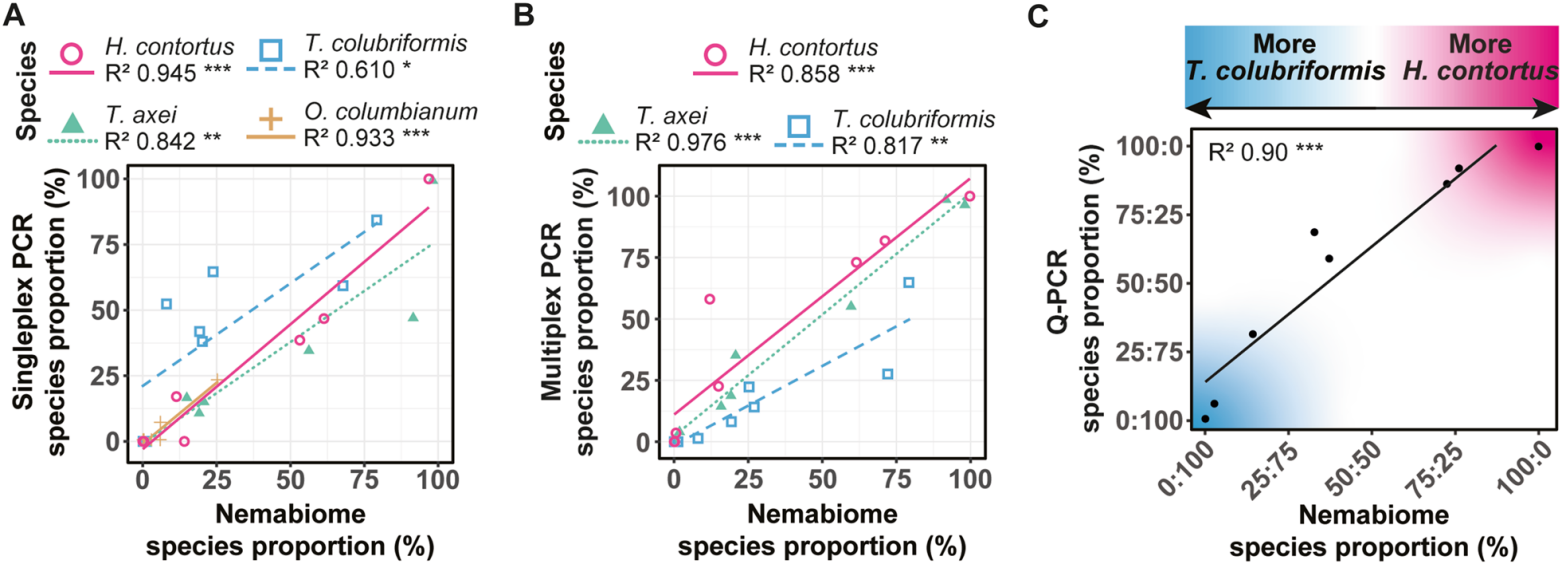
Nemabiome validation of quantitative PCR and HRMC analyses. Cross-validation of PCR methods to nemabiome species proportions normalised to number of species tested including **A** singleplex semi-quantitative PCR with four species, **B** multiplex semi-quantitative PCR with three species, and **C** qPCR with two species. *P < 0.05, **P < 0.01, ***P < 0.001

To validate PCR strategies used, species proportions were cross-correlated between quantitative PCR approaches and nemabiome results using the Mothur pipeline (Fig. 7) or the DADA2 pipeline with IDTAXA (Additional file 8: Fig. S5). All species predicted by PCR were significantly correlated to both nemabiome pipelines. The main differences in relative detection were associated with *T. colubriformis* which could be skewed due to differences in *Trichostrongylus* read classification between pipelines. Predictive proportions were most accurate for *H. contortus* for singleplex PCR (Fig. 7A) and *T. axei* for multiplex PCR (Fig. 7B; Additional file 8: Fig. S5A). qPCR was also highly correlated to both nemabiome pipelines but somewhat overestimated *T. colubriformis* abundance (Fig. 7C; Additional file 8: Fig. S5B).

## Discussion

At the current time, the utility of molecular techniques for monitoring veterinary parasites is limited in low-resource subsistence farming areas as well as in most commercial livestock settings [1–3]. In the present study, we demonstrate the utility of combining low-resource GIN monitoring techniques (FPC and FECs) with molecular diagnostics on heat-desiccated preserved faeces and explore molecular options to determine the presence and abundance of different parasite species. In-depth molecular assessment of GINs typically requires culture and isolation of L3 larvae, which can then be preserved in ethanol [47], but eggs and L1 larvae are also sufficient for analysis and can avoid potential biases in larval hatching [49]. However, extraction and purification of eggs or larvae takes time and requires fresh faecal samples. Rural communities and resource-poor areas may not have the capacity to process fresh samples and may also lack immediate access to clean ethanol or other preservatives which do not interfere with DNA quality. To develop a protocol which is as amenable as possible to rural settings, we turned to heat desiccation, which can be performed via a number of chemical- and electrical-free means such as by sunlight or heating over a low flame.

Desiccation is a proven means of preserving DNA from faeces [64] and blood [65], but has yet to be utilised to measure GINs. Faecal DNA extractions have been shown to predict infection burden [32–34] and detect as low as 500 fg of parasite DNA [35] or 10 EPG [33] and can be more sensitive than FEC for detection and quantitation of GIN infections [35]. In this study, GINs could be identified from all individuals, demonstrating that parasite DNA is preserved effectively by desiccation (see Fig. 2). Since these are natural infections, determining the limit of parasite detection was not possible, but a correlation was identified between FEC and the number of strongyle species detected (see Additional file 8: Fig. S2A, B).

Collection of faecal material can facilitate biobanking, but low-cost and low-resource methods are also needed to enable the study of goat health in developing countries. Molecular diagnostics differ drastically in cost, throughput, and ability to quantify outputs (see Additional file 2: Table S2; Additional file 4: Table S4). To this end, this study compared low-resource magbead and high-resource spin-column extraction methods, with the high-resource method yielding higher-quality DNA (Additional file 8: Fig. S1B). Despite differences in resources required, there were no noticeably different results regarding species identification among different DNA extraction methods used on the same goat population (Additional file 8: Fig. S2C). Since there were considerable differences in overall costs among the methods tested, the low-resource method produces satisfactory results for cases when the high-resource method is out of reach. The use of paramagnetic beads for DNA extraction from difficult samples also shows promise for ‘kit-free’ approaches [66]. However, the low-resource method did require centrifugation in some cases to remove carryover contaminants and would also require additional quality control steps in field or remote lab settings (Additional file 8: Fig. S1C, D).

Profiling co-infection species composition typically requires advanced and costly molecular methods [34, 45, 47]. While standard endpoint PCR is conventionally considered insufficient for relative quantification of PCR targets, we identify that endpoint and simple semi-quantitative approaches closely mirror qPCR findings (Fig. 4), while being more amenable to increased throughput at a lower cost. Both the singleplex and multiplex semi-quantitative approaches can close the gap for molecular detection of parasites and other diseases in cost-limited farming systems. Overall, semi-quantitative multiplex PCR was the most promising method, as multiplexing increased throughput, reduced reagent costs, and was the method most comparable to quantitative and sequencing results. However, in this study we were limited by the number of different target species detectable per multiplex reaction. Issues of throughput regarding number of possible target species could be overcome through improved separation of PCR products such as by capillary electrophoresis, which is amenable to low-cost and field-applicable labs [67]. In rural or field settings, PCR is achievable from solar or battery packs [68, 69] and can avoid cold-chain limitations when using freeze-dried reagents [70]. Detection of PCR products can also forgo electrophoresis entirely in settings with limited electricity through DNA dipsticks, which are nucleic acid lateral flow assays that can be used to detect specific species through modified probes [71–73]. There are other, more direct alternatives available including LAMP assays which have been optimised for GINs and have great promise for field settings [38, 39]. Whether dipstick or LAMP methods will be cost-effective compared to standard PCR to detect residual abundance of helminth infections requires further optimisation.

Validating PCR results by nemabiome deep amplicon sequencing provided the first attempt to use this platform to assess GIN infections in African small ruminants and revealed infections to be dominated by *H. contortus* in the majority of individuals, but some individuals were potentially more heavily infected with *T. axei* or *T. colubriformis*. Interestingly, performing nemabiome on desiccated faecal samples resulted in high-quality reads and as such, DNA degradation was minimal following desiccation and preservation. This method may prove useful to survey wildlife faecal samples when chemical preservatives are out of reach. Overall, nemabiome results mirrored PCR results and did not reveal any other highly present GIN species. As such, semi-quantitative PCR may be a sufficient route to determine parasite populations in low-resource settings. It should be noted that nemabiome style amplicon sequencing can offer distinct advantages over species-specific PCR, principally the capacity to detect novel and unaccounted-for species that may be present in a sample. However, the cost of performing amplicon sequencing will always be far more expensive than PCR alone, since amplicon sequencing relies on initial production of amplicons through multiple rounds of PCR, in addition to PCR product clean-up, quality control steps, library preparation, sequencing, and downstream bioinformatics analyses.

In addition to PCR and qPCR techniques, HRMC analyses were performed using pan-nematode ITS-2 primers which should amplify strongyle GIN species equally and avoid biases such as primer binding and PCR product size impact that can occur between primer sets and probes on mixed DNA samples. HRMC can also provide a semi-quantitative picture of parasitic nematode identification when PCR products from different species have distinct melting temperatures due to sequence variation [41, 74]. HRMC has recently been utilised to distinguish *Trichostrongylus* species [40], as well as *H. contortus* alongside other GINs from sheep [41]. In this study, we identify distinct melt curves for high *H. contortus* and *T. colubriformis* samples (see Additional file 5: Table S5), but the addition of other species or mixes of species resulted in melt temperature shifts which could not be rationally deciphered in this dataset as compared to more controlled studies. While HRMC is more expensive than traditional PCR techniques, it can save costs overall by reducing tube numbers required for the detection of different species compared to qPCR (see Table 1).

The use of field-applicable techniques to monitor goat health and parasite burden can have lasting impacts on sustainable livestock farming systems. Localised goat breeds are an invaluable asset to global food security, but determining co-infection burden along with GIN species composition is essential to understand the potential limiting impacts of parasites on local breed production and guide intervention decisions. This is valuable considering GIN species have differential impacts on goat heath. For instance, the hematophagous *H. contortus* is highly fecund and can cause anaemia and death in heavily infected or susceptible small ruminants [31, 75], while *O. columbianum* is a far less abundant species, but can cause scour and weight loss resulting from intestinal nodules [22]. Other GINs such as *Trichostrongylus* species can damage intestinal walls resulting in enteritis and loss of protein through haemorrhage of the mucosal lining, mirroring signs of malnutrition [76, 77]. Knowledge of the main GIN species present and variation in species composition between individuals and across seasons can underpin optimised monitoring systems, for example by simplifying the FPC to focus on dominant GIN-driven pathologies, and/or support adaptive breed improvement strategies. In some regions, breeding programs have focused on improved resistance or resilience to GIN species [26, 78, 79]. However, in Malawi, goats can and often do succumb to GIN infections (despite being resilient in comparison to Boer and other exotic breed goats) with up to 80% of younger adult goats dying due to diseases including helminthiasis [20, 21]. Development of GIN-resilient and/or GIN-resistant breeds requires careful monitoring of GIN presence and co-infection, but the technology to characterise GIN co-infection burden is limited in rural areas. Monitoring of GIN populations in Malawi and other low-income areas will be bolstered significantly by the introduction of molecular methods to determine the spread and abundance of different GIN species. This is especially important since FPC and FEC offer a generalised view of the GIN infection intensity or burden of infection [27, 37] but are unable to determine the relative abundance of different species present by egg morphology alone. As such, monitoring of specific species can aid monitoring and breeding programs alike, and also improve the accuracy of tests for anthelmintic drug resistance based on FEC, which are confounded by mixed species infections [80]. The use of a single pellet sample limits the amount of faeces required while fairly reflecting overall egg output [81], whereas preservation by desiccation and use of conventional PCR technology greatly improves the accessibility of the method, especially in remote or rudimentary settings. Overall, chemical-free biobanking of samples with later DNA extraction and low-cost PCR diagnostics will enable monitoring not only of goat GINs but potentially of other parasitic helminths in remote settings.

## Conclusions

We find that heat-desiccated faeces not only retains high-quality DNA but also enables quantitative detection of various parasitic species by PCR, qPCR, HRMC, and deep amplicon sequencing (nemabiome) analyses. Lower-cost semi-quantitative methods also highly correlate to more expensive methods and may prove valuable for use in species-specific diagnostics from rural or low-resource biobanked samples. Low-cost techniques can also link GIN co-infection compositions to health checks and goat performance data to identify underlying causes of livestock losses. Given the diverse nature of infections in livestock and wildlife, further study is needed to tailor these methods for the surveillance and monitoring of parasites and possibly host genetic traits in rural areas.

### Supplementary Information

**Additional file 1. Table S1.** Raw baseline data on DNA extraction, quality, quantity and yield

**Additional file 2. Table S2. **Primers and cycling conditions used for each PCR technique

**Additional file 3. Table S3. **Nemabiome raw read results from Eurofins 2nd PCR amplicon sequencing

**Additional file 4. Table S4. **DNA extraction costs

**Additional file 5. Table S5. **HRMC peak temperatures and Singleplex PCR relative species abundance

**Additional file 6. Table S6. **Proportional read differences between Mothur and DADA2 nemabiome pipelines

**Additional file 7. File S1. **72-well gel electrophoresis comb design

**Additional file 8. Figures S1–S5. Figure S1:** Assessing quality of DNA extraction methods. **Figure S2:** Assessing GIN species detection likelihoods by DNA extraction method and infection intensity. **Figure S3:** High-resolution melt curves with pan-nematode ITS-2 primers. **Figure S4:** Nemabiome ITS-2 amplicon with the DADA2 IDTAXA pipeline. **Figure S5:** Cross-validation of PCR methods with nemabiome using the DADA2 IDTAXA pipeline.

## Data Availability

Raw diagnostic, DNA extraction, and PCR data are shown in Additional file 1: Table S1. All other original results including tabulated data, PCR results, and other materials are available on request where not included in the manuscript.

## Abbreviations

BCS: Body condition score
EPG: Eggs per gram
FAMACHA: Faffa Malan chart
FEC: Faecal egg count
FPC: Five Point Check^©^
GIN: Gastrointestinal nematode
HRMC: High-resolution melt curve
ITS-2: Internal transcribed spacer 2
LAMP: Loop-mediated isothermal amplification
NMDS: Non-metric multidimensional scaling
OPG: Oocysts per gram
PCR: Polymerase chain reaction
qPCR: Quantitative polymerase chain reaction
