# Supplementary material for: Low-cost molecular methods to characterise gastrointestinal nematode co-infections of goats in Africa
Source: Parasit Vectors. 2023 Jun 29;16:216. doi: 10.1186/s13071-023-05816-y (PMC10311829; doi:10.1186/s13071-023-05816-y)
Supplement: Supplementary file 8 — Additional file 8. Figures S1–S5. Figure S1: Assessing quality of DNA extraction methods. Figure S2: Assessing GIN species detection likelihoods by DNA extraction method and infection intensity. Figure S3: High-resolution melt curves with pan-nematode ITS-2 primers. Figure S4: Nemabiome ITS-2 amplicon with the DADA2 IDTAXA pipeline. Figure S5: Cross-validation of PCR methods with nemabiome using the DADA2 IDTAXA pipeline. [file 13071_2023_5816_MOESM8_ESM.docx]

# Supplementary Materials for

# “Low-cost molecular methods to characterise gastrointestinal nematode co-infections of goats in Africa”

## Authors:

Paul M Airs^1^, Javier Ventura-Cordero^1^, Winchester Mvula^2^, Taro Takahashi^3,4^, Jan Van Wyk^5^, Patson Nalivata^2^, Andrews Safalaoh^2^, Eric R Morgan^1*^

**Affiliations:**

1. School of Biological Sciences, Queen’s University Belfast, Belfast, Antrim, United Kingdom
2. Animal Science Department, Lilongwe University of Agriculture and Natural Resources (LUANAR), Lilongwe, Malawi
3. Net Zero and Resilient Farming Directorate, Rothamsted Research, Okehampton, Devon, United Kingdom
4. Bristol Veterinary School, University of Bristol, Langford, Somerset, United Kingdom
5. Department of Veterinary Tropical Diseases, University of Pretoria, Pretoria, South Africa

# Supplementary Figures


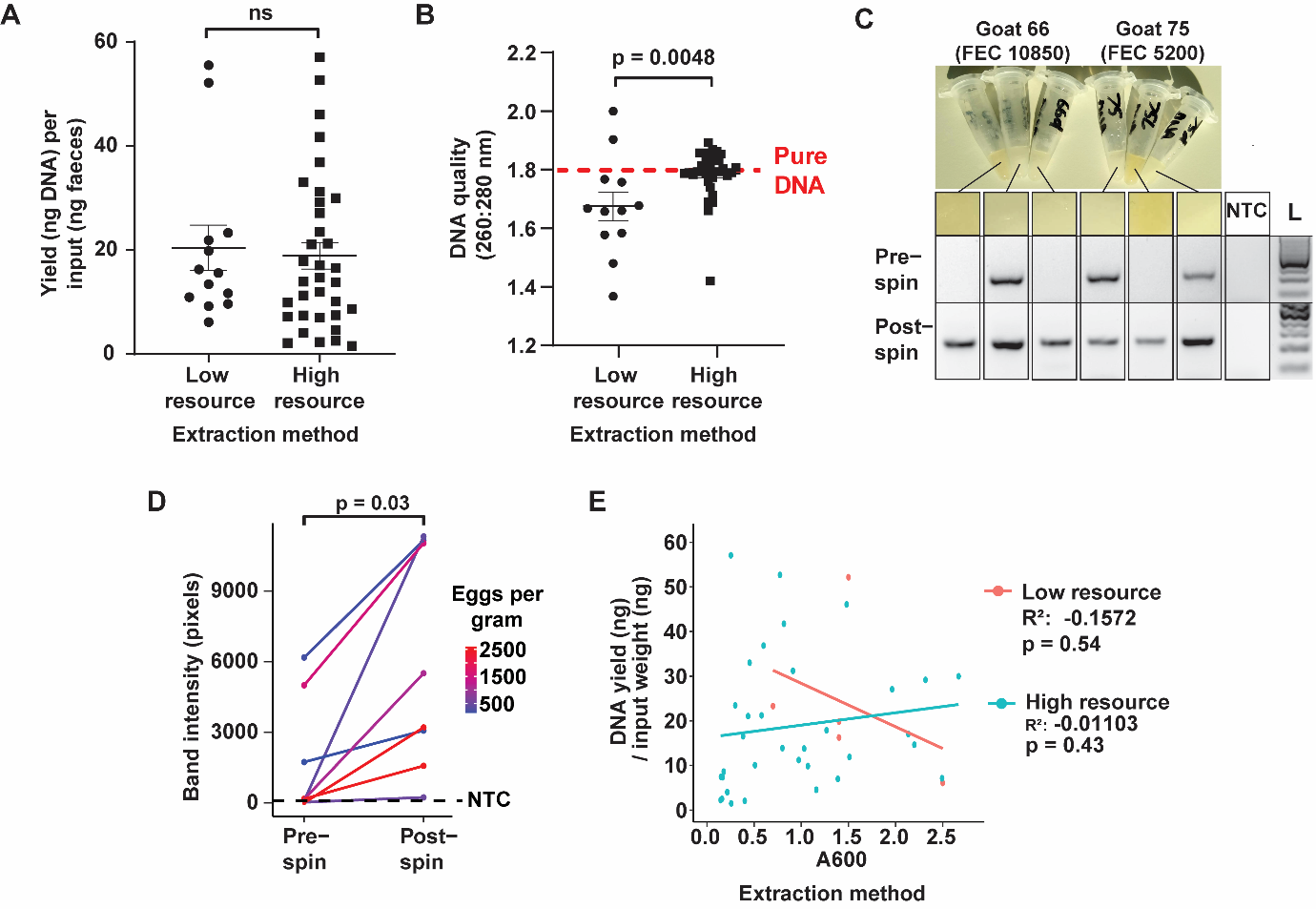


## Fig. S1: Assessing quality of DNA extraction methods.

Differences in low vs high-resource methods were investigated including (A) yield per input weight of faeces and (B) DNA purity by spectroscopy. (C-D) For the low-resource kit faecal contamination carryover resulted in PCR inhibition in some extractions, but could be remedied by centrifugal pelleting of contaminants. (E) Coloration of faecal homogenates, a measure of faecal content and resuspension following desiccation, did not impact DNA yield.


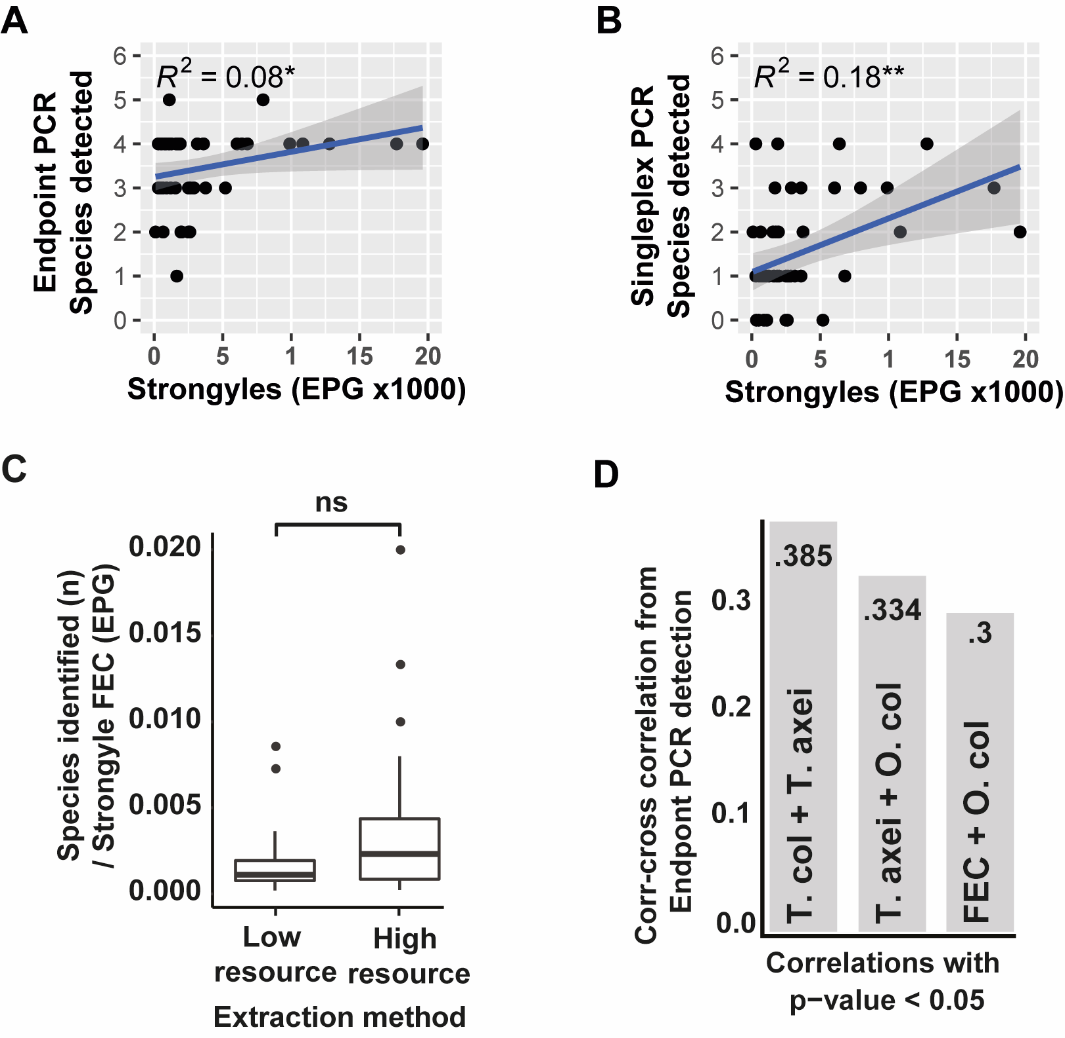


## Fig. S2: Assessing GIN species detection likelihoods by DNA extraction method and infection intensity.

(A-B) Regression of Strongyle GIN species identified per goat sample by Strongyle EPG using (A) Endpoint PCR detection and (B) Singleplex semi-quantitative PCR detection. (C) GIN species detected between low-resource and high-resource DNA extraction methods normalised to Strongyle EPG per sample, compared by student's T-test. (D) Ranked cross-correlations for different individual species identified and FEC from endpoint PCR results in all samples. O. col = *O. columbianum*, T. col = *T. colubriformis*, Ns = not significant, * = P < 0.05, ** = P < 0.01.


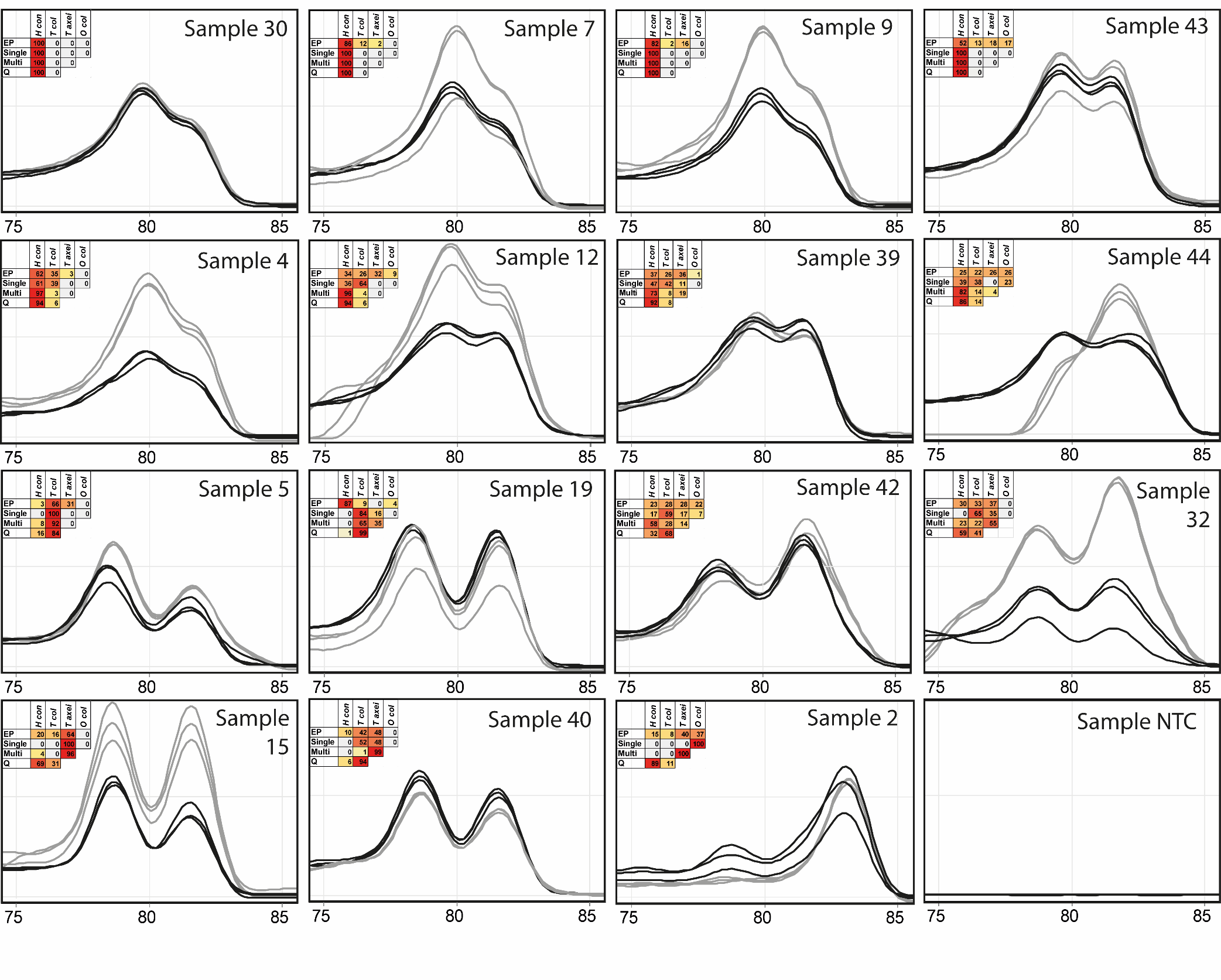


## Fig. S3: High resolution melt curves with pan-nematode ITS-2 primers.

All melt curves with replicate 1 (grey lines) and replicate 2 (black lines) shown. Inserts show relative species abundance (as percentage) from EP = endpoint PCR, single = Singleplex PCR, multi = Multiplex PCR, and Q = Q-PCR analyses. NTC = No template control. Some sample peaks differ in height due to template dilutions between replicates being required to yield sufficient sample volume for the second replicate.


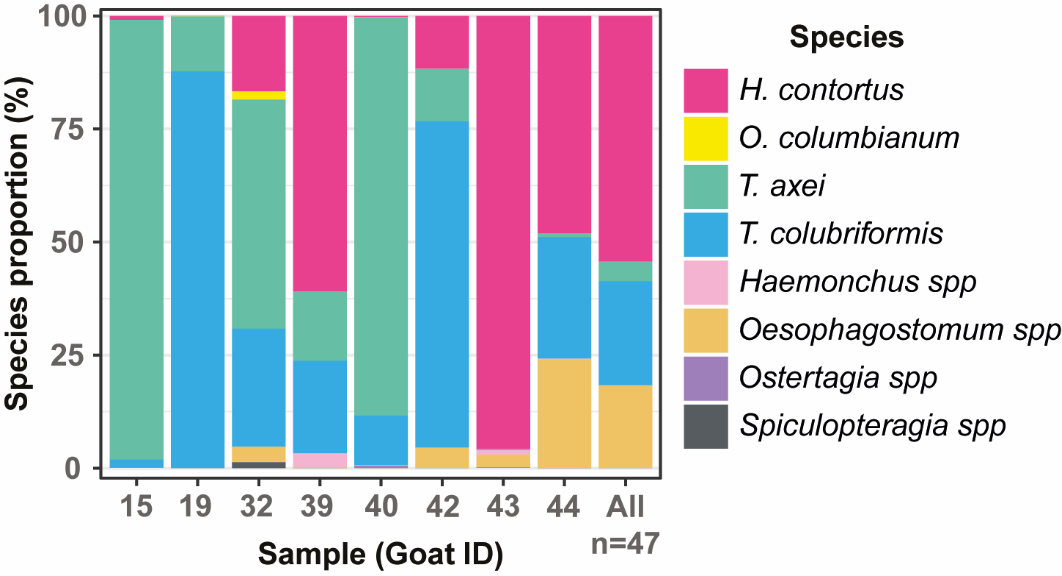


## Fig. S4: Nemabiome ITS-2 amplicon with the DADA2 IDTAXA pipeline.

DADA2 resolved proportional GIN species and unresolved species from select goat samples as well as pooled DNA from all goat samples.


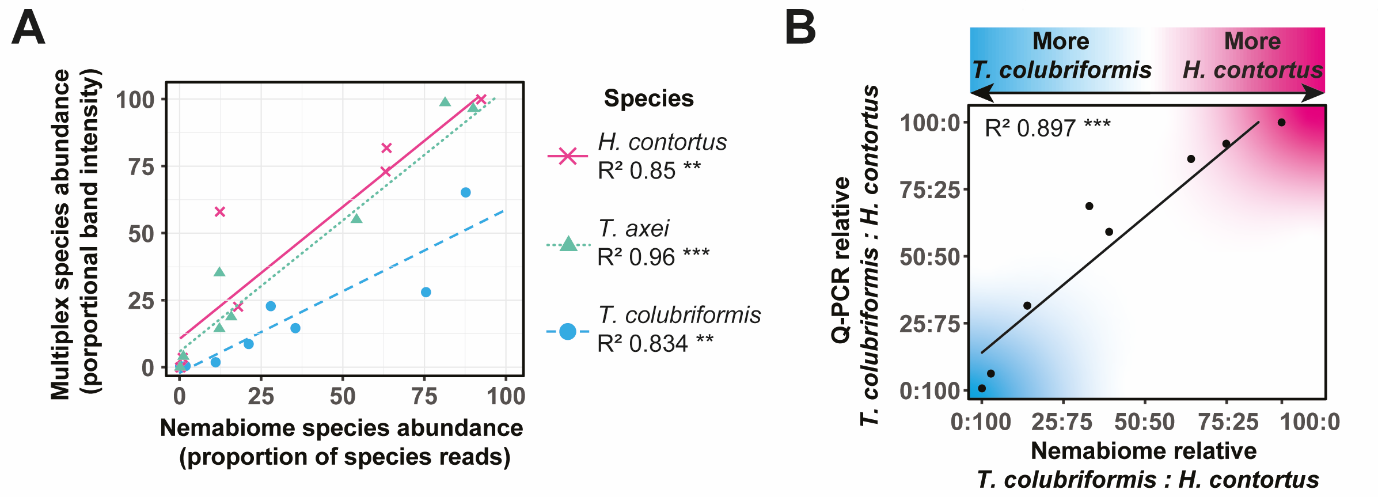


## Fig. S5: Cross-validation of PCR methods with Nemabiome using the DADA2 IDTAXA pipeline.

Cross-validation of PCR methods to Nemabiome species proportions normalised to number of species tested including (A) multiplex semi-quantitative PCR with three species, and (B) Q-PCR with two species. ** = P < 0.01, *** = P <0.001.

# Supplementary Tables

[file uploaded separately]

## Table S1: Raw baseline data

[file uploaded separately]

## Table S2: Primers and cycling conditions used for each PCR technique

* Bold primers are pan-nematode, italicised primers were used in multiplex PCR

† PCR methods include (EP) endpoint standard singleplex PCR, (SP) singleplex semi-quantitative PCR, (MP) multiplex endpoint semi-quantitative PCR, (Q) Q-PCR, (HMC) Melt-curve analysis.

[file uploaded separately]

## Table S3: Nemabiome raw read results from Eurofins 2nd PCR amplicon sequencing

[file uploaded separately]

## Table S4: DNA extraction costs

The high-resource and low-resource methods compared in terms of costs per extraction including the kit costs at the time of the study and associated costs for essential equipment. Consumables such as pipette tips and labour costs are not included here.

[file uploaded separately]

## Table S5: HRMC peak temperatures and Singleplex PCR relative species abundances.

Peak temperature averages and standard deviation (SD) from 6 technical replicates aligned to singleplex PCR species relative abundance data (as percentage).

[file uploaded separately]

## Table S6: Proportional read differences between Mothur and DADA2 Nemabiome pipelines

# Supplementary Files

[file uploaded separately]

## File S1: 72 well gel electrophoresis comb design.

TinkerCAD designed file for production of acrylic laser cut comb for multichannel pipette fast-loading and high-throughput gel electrophoresis.
